# EXPLORING DEI IN A GLOBAL PERSPECTIVE: AGHE INSIGHTS INTO OUR WORK SUPPORTING GERONTOLOGY AND GERIATRIC EDUCATORS

**DOI:** 10.1093/geroni/igac059.868

**Published:** 2022-12-20

**Authors:** Dana Bradley, Judith Howe

**Affiliations:** University of Maryland, Baltimore County, Baltimore, Maryland, United States; Ichan School of Medicine at Mount Sinai, Bronx, New York, United States

## Abstract

Gerontologists across the globe make a sincere effort to create a learning, teaching, and research space that promotes the dignity of older persons. Indeed, this impetus imbues our work where all are treated with dignity and respect. The authors (AGHE chair and past chair) reached out to international educators to better understand how AGHE is supporting their efforts and how we may do so in the future. Our starting point was the GSA policy on Diversity, Equity, and Inclusion (2021). These policies laid out a groundwork that embraces inclusive membership cultivates practices that build a culturally inclusive workforce, foster research practices, and ensure that people from disproportionately affected and marginalized communities thrive in GSA. This paper offers insight into how our international community is thinking about DEI and incorporating it into teaching, research and engagement work.

